# Association of serum creatinine levels and risk of type 2 diabetes mellitus in Korea: a case control study

**DOI:** 10.1186/s12902-021-00915-2

**Published:** 2022-01-04

**Authors:** Do Kyeong Song, Young Sun Hong, Yeon-Ah Sung, Hyejin Lee

**Affiliations:** Department of Internal Medicine, Ewha Womans University School of Medicine, 25 Magokdong-ro 2-gil, Gangseo-gu, Seoul, 07804 South Korea

**Keywords:** Creatinine, Type 2 diabetes mellitus, Risk

## Abstract

**Background:**

Reduced skeletal muscle has been suggested as a potential risk factor for type 2 diabetes mellitus (T2DM). Serum creatinine is the primary metabolite of creatine in skeletal muscle. Therefore, low serum creatinine levels may be associated with an increased risk of T2DM. We aimed to evaluate the association between serum creatinine levels and the risk of T2DM in Korea.

**Methods:**

We analyzed a total of 264,832 nondiabetic adults older than 40 years of age who had undergone a national health examination at least once from 2009 to 2015 in the Korean National Health Insurance Service Cohort. Hazard ratios for T2DM were calculated.

**Results:**

In men, serum creatinine levels and the risk for T2DM showed an inverse J-shaped association. This association was confirmed after adjustment for age, body mass index (BMI), systolic blood pressure (SBP), diastolic blood pressure (DBP), and fasting plasma glucose. In women, there was a trend that serum creatinine levels were inversely associated with the risk of T2DM among those with serum creatinine below 1.1 mg/dl. However, serum creatinine levels were not significantly associated with the risk of T2DM after adjustment for age, BMI, SBP, DBP, and fasting plasma glucose.

**Conclusions:**

Reduced levels of serum creatinine were significantly associated with an increased risk of T2DM in men with creatinine below 1.20 mg/dl. There was a trend that decreased levels of serum creatinine were associated with an increased risk of T2DM among women with serum creatinine below 1.1 mg/dl, although this result was not statistically significant.

## Background

Skeletal muscle is the major target tissue for insulin-mediated glucose uptake and peripheral insulin resistance (IR) [1, 2]. Therefore, muscle loss may result in a reduction in targets for insulin action and the generation of IR. Increased IR associated with low muscle mass may induce the development of diabetes. Reduced muscle mass was reported to be associated with increased IR among subjects aged 20 years or older by the National Health and Nutrition Examination Survey III data [3]. In turn, greater thigh muscle mass was associated with a lower risk of incident type 2 diabetes mellitus (T2DM) for leaner Japanese Americans without diabetes [4]. In Korean subjects ≥20 years of age, reduced skeletal muscle mass measured using dual energy X-ray absorptiometry was associated with increased IR and was a risk factor for diabetes in the nonobese population based on the Korea National Health and Nutritional Examination Survey [5]. A prospective study including middle-aged and older Korean adults without diabetes showed that reduced muscle mass was associated with an increased risk of T2DM for a given body mass index (BMI) category [6].

Serum creatinine is primarily a breakdown product of creatine that depends on the total skeletal muscle mass and is consistently eliminated in the steady state. Although serum creatinine is known to be influenced by age, sex, ethnicity, and dietary protein intake, serum creatinine is very stable and often used as an easily measured surrogate marker of skeletal muscle mass when protein intake is adequate [7, 8]. Therefore, there is the potential that low serum creatinine is associated with the development of T2DM. Because measuring serum creatinine levels is less expensive and easier than measuring the amount of skeletal muscle mass, evaluating the association between serum creatinine levels and the incidence of T2DM is interesting and useful for the early detection of high-risk T2DM.

Several studies have demonstrated that low serum creatinine levels are associated with an increased risk of diabetes. A prospective cohort study in a general population sample from China showed that the serum creatinine concentration was inversely related to incident T2DM similarly for both men and women [9]. A cross-sectional study including morbidly obese Caucasian patients with an estimated glomerular filtration rate > 60 ml/min/1.73 m^2^ showed that low serum creatinine was a predictor of type 2 diabetes independent of age, sex, family history of diabetes, hypertension, and current smoking [10]. Additionally, in Korea, a study including 2676 subjects without diabetes (estimated glomerular filtration rate > 60 mL/min/1.73 m^2^) showed that a decrease in serum creatinine during the follow-up period was associated with an increased risk of T2DM, although baseline serum creatinine levels were not different between subjects with new-onset diabetes and those without diabetes [11]. However, because the previous study was performed with small numbers of participants in a single center, it is difficult to generalize the results of the study in Korea. Therefore, we aimed to evaluate the associations between the levels of serum creatinine and the risk of incident T2DM in a Korean population older than 40 years using a large cohort database based on Korean National Health Insurance Service (NHIS) data between 2009 and 2015.

## Methods

### Data source

We analyzed the cohort database released by the Korean NHIS from 2009 to 2015. Since the Korean National Health Insurance Program is a universal health insurance program in Korea and approximately 97% of the Korean population is enrolled in the NHIS, the cohort can represent the Korean general population. The Korean NHIS was initiated in 1963 and has been described in detail elsewhere [12]. All members of the population older than 40 years are invited to participate in a biannual health checkup as part of the NHIS. The participants completed a self-administered questionnaire including information on demographic, medical, and behavioral factors.

We did not obtain informed consent from the participants because the data were not collected for the study. The patient records from the NHIS were anonymous before being released by the NHIS. This study was approved by the Institutional Review Board of Ewha Medical Center. All methods were performed in accordance with the relevant guidelines and regulations.

### Study population & outcome variables

From 2009 to 2015, subjects older than 40 years who had received at least one health examination were enrolled. Among them, subjects who had abnormally high levels of serum creatinine or with missing data were excluded. We further excluded subjects with a self-reported history of diabetes or fasting plasma glucose ≥126 mg/dl.

Serum creatinine is known to be influenced by sex, and women tend to have lower levels of serum creatinine [8]. Therefore, we divided serum creatinine into four categories for men and women differently [13, 14]. For men, the categories were < 0.7, 0.7–0.89, 0.9–1.19, and ≥ 1.2 mg/dl; for women, the categories were < 0.6, 0.6–0.79, 0.8–1.09, and ≥ 1.1 mg/dl taking into account the serum creatinine distribution in our study and serum creatinine cut-off points used in previous studies [13, 15]. The reference serum creatinine was below 0.7 mg/dl for men and below 0.6 mg/dl for women.

The primary outcome was the incidence of T2DM. Using the definition of diabetes based on the American Diabetes Association criteria, T2DM was defined as having fasting plasma glucose ≥126 mg/dl or glycated hemoglobin (HbA1c) ≥ 6.5% or receiving hypoglycemic agents [16].

### Statistical analyses

The Kolmogorov-Smirnov statistic was used to analyze continuous variables for normality. Data are shown as the means ± standard deviations for continuous variables and frequency (percentage) for categorical variables. We used Cox proportional hazards models to calculate the crude and adjusted hazard ratios (HRs) and 95% confidence intervals for estimating the risk of diabetes associated with serum creatinine levels. In model 1, HRs were calculated without adjusting for covariates. Model 2 was adjusted for age and BMI. Model 3 was adjusted for age, BMI, systolic blood pressure (SBP), diastolic blood pressure (DBP), and fasting plasma glucose. The figures were modeled using predicted HR values for each creatinine level.

The analyses were conducted separately for men and women. *P* values < 0.05 were considered statistically significant. All statistical analyses were performed using SAS (version 9.4, SAS Institute, Cary, NC).

## Results

From 2009 to 2015, a total of 514,866 subjects (> 40 years) had received at least one health examination. Among them, 69,067 who had abnormally high levels of serum creatinine (> 1.4 mg/dl) or were missing data on blood pressure, blood glucose, BMI, or creatinine and 180,967 patients with a self-reported history of diabetes or fasting plasma glucose ≥126 mg/dl were excluded. Finally, a total of 264,832 subjects (139,109 men and 125,723 women) were included in the study.

**Fig. 1:**
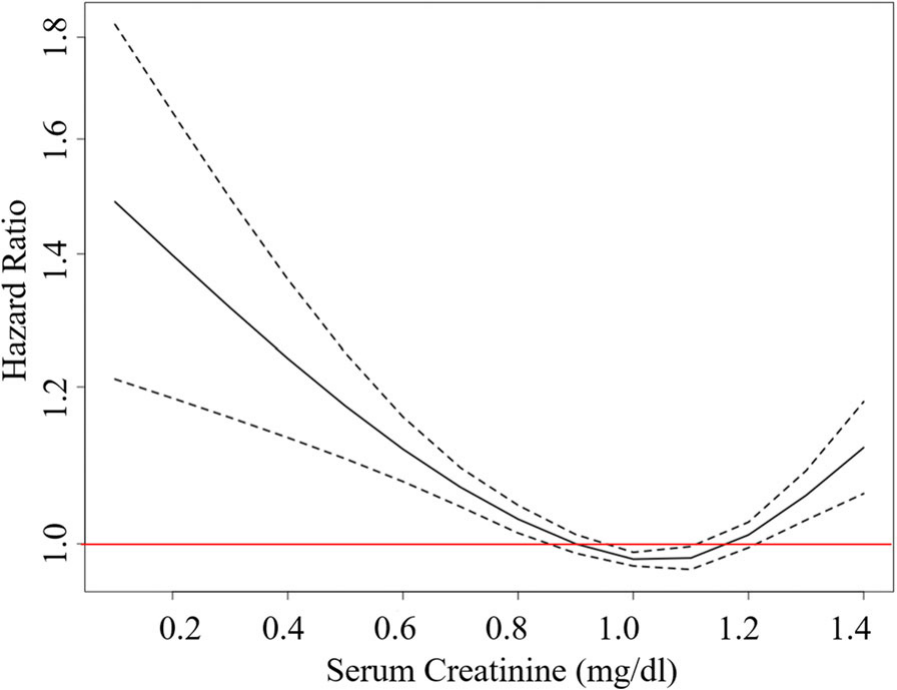
Adjusted hazard ratios of incident diabetes by creatinine levels in men. Hazard ratios were adjusted for age, body mass index, systolic blood pressure, diastolic blood pressure, and fasting plasma glucose

During the follow-up period, 32,209 men and 32,965 women had been diagnosed with T2DM. Table 1 shows the basal characteristics of men according to the baseline serum creatinine categories. Men with increased serum creatinine levels tended to have increased mean BMI, DBP, and fasting plasma glucose. In men, serum creatinine levels and the risk for T2DM showed an inverse J-shaped association, with the lowest risk among men with serum creatinine between 0.90 and 1.19 mg/dl (Fig. 1). This inverse J-shaped association between serum creatinine levels and the risk for T2DM was confirmed after adjustment for age, BMI, SBP, DBP, and fasting plasma glucose. Men with the lowest serum creatinine had the highest risk of incident T2DM. The HRs of diabetes incidence by creatinine levels in men are shown in Table 2. The hazard ratios for the development of T2DM according to the serum creatinine categories were 1.00 (reference), 0.90 (0.7–0.89 mg/dl), 0.88 (0.9–1.19 mg/dl), and 0.95 (≥ 1.2 mg/dl) after age, BMI, SBP, DBP, and fasting plasma glucose in men.

**Table 1:** Basal clinical characteristics of male participants according to the serum creatinine categories

	Serum creatinine (mg/dl)				*P* for trend
	< 0.7	0.7–0.89	0.9–1.19	≥ 1.2	
n (%)	9689 (7.0)	44,054 (31.7)	76,779 (55.2)	8587 (6.2)	
Age (years)	59 ± 9	58 ± 8	57 ± 8	58 ± 9	< 0.001
BMI (kg/m^2^)	23.3 ± 2.8	23.6 ± 2.8	24.1 ± 2.7	24.5 ± 2.7	< 0.001
SBP (mmHg)	126 ± 15	126 ± 15	125 ± 15	126 ± 15	< 0.001
DBP (mmHg)	78 ± 10	79 ± 10	79 ± 10	79 ± 10	0.034
FPG (mg/dl)	94 ± 12	95 ± 11	95 ± 11	95 ± 12	< 0.001
Mean follow up period (years)	4.7 ± 1.9	4.8 ± 1.8	4.9 ± 1.8	4.8 ± 1.9	< 0.001

*BMI* body mass index, *SBP* systolic blood pressure, *DBP* diastolic blood pressure, *FPG* fasting plasma glucose

**Table 2:** Associations between serum creatinine and incidence of diabetes in men

	Serum creatinine (mg/dl)				*P* for trend
	< 0.7	0.7–0.89	0.9–1.19	≥ 1.2	
Incident diabetes, n (%)	2476 (25.6)	9981 (22.7)	17,493 (22.8)	2259 (26.3)	< 0.001
Model 1	Reference	0.86 (0.83, 0.90)	0.85 (0.81, 0.88)	1.00 (0.95, 1.06)	
Model 2	Reference	0.90 (0.86, 0.94)	0.89 (0.85, 0.92)	0.96 (0.91, 1.02)	
Model 3	Reference	0.90 (0.86, 0.94)	0.88 (0.85, 0.92)	0.95 (0.90, 1.01)	

Model 1: unadjusted. Model 2: adjusted for age and body mass index. Model 3: adjusted for age, body mass index, systolic blood pressure, diastolic blood pressure, and fasting plasma glucose

**Fig. 2:**
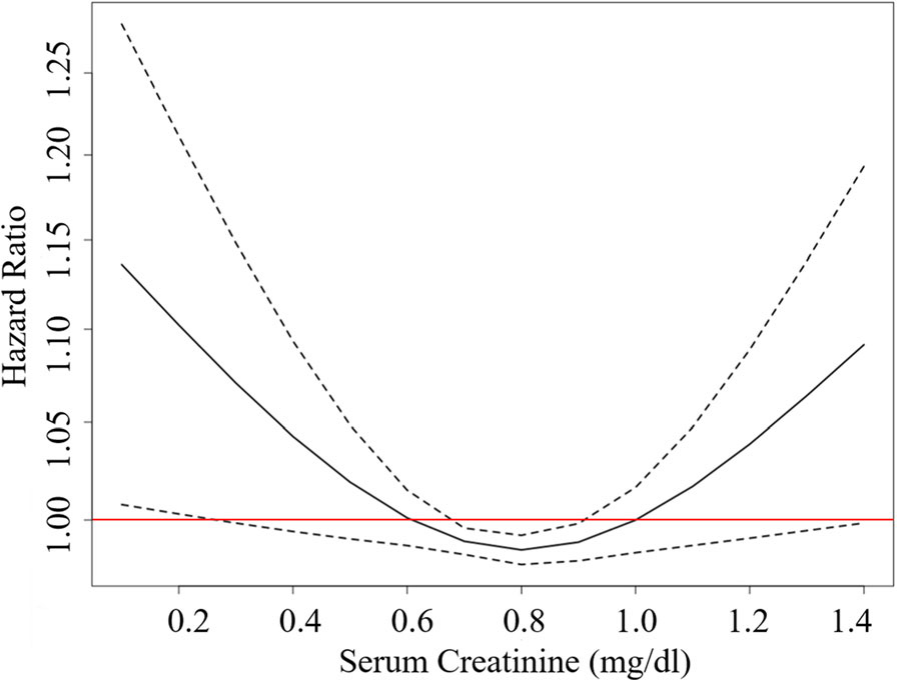
Adjusted hazard ratios of incident diabetes by creatinine levels in women. Hazard ratios were adjusted for age, body mass index, systolic blood pressure, diastolic blood pressure, and fasting plasma glucose

Table 3 shows the basal characteristics of women according to the baseline serum creatinine categories. Women with increased serum creatinine levels tended to have increased mean BMI, DBP, and fasting plasma glucose. In women, there was a trend that reduced levels of serum creatinine were associated with an increased risk of T2DM among those with serum creatinine below 1.1 mg/dl, although the association was not statistically significant (Fig. 2). Women with the highest serum creatinine levels had the highest risk of T2DM. However, serum creatinine levels were not significantly associated with the risk of T2DM after adjustment for age, BMI, SBP, DBP, and fasting plasma glucose in women. The HRs of diabetes incidence by creatinine levels in women are shown in Table 4. The hazard ratios for the development of T2DM according to the serum creatinine categories were 1.00 (reference), 0.99 (0.6–0.79 mg/dl), 0.98 (0.8–1.09 mg/dl), and 1.06 (≥ 1.1 mg/dl) after age, BMI, SBP, DBP, and fasting plasma glucose in women.

**Table 3:** Basal clinical characteristics of female participants according to the serum creatinine categories

	Serum creatinine (mg/dl)				*P* for trend
	< 0.6	0.6–0.79	0.8–1.09	≥ 1.1	
n (%)	23,080 (18.4)	65,760 (52.3)	35,249 (28.0)	1634 (1.3)	
Age (years)	58 ± 9	58 ± 8	59 ± 9	64 ± 11	< 0.001
BMI (kg/m^2^)	23.5 ± 3.0	23.6 ± 2.9	23.8 ± 3.0	24.1 ± 3.2	< 0.001
SBP (mmHg)	123 ± 16	122 ± 15	123 ± 16	126 ± 16	< 0.001
DBP (mmHg)	76 ± 10	76 ± 10	76 ± 10	77 ± 10	< 0.001
FPG (mg/dl)	92 ± 11	93 ± 10	93 ± 11	95 ± 12	< 0.001
Mean follow up period (years)	4.7 ± 1.8	4.8 ± 1.8	4.8 ± 1.8	4.5 ± 2.0	< 0.001

*BMI* body mass index, *SBP* systolic blood pressure, *DBP* diastolic blood pressure, *FPG* fasting plasma glucose

**Table 4:** Associations between serum creatinine and incidence of diabetes in women

	Serum creatinine (mg/dl)				*P* for trend
	< 0.6	0.6–0.79	0.8–1.09	≥ 1.1	
Incident diabetes, n (%)	5923 (25.7)	16,938 (25.8)	9545 (27.1)	559 (34.2)	< 0.001
Model 1	Reference	0.98 (0.95, 1.01)	1.03 (0.99, 1.06)	1.40 (1.29, 1.53)	
Model 2	Reference	0.99 (0.96, 1.02)	1.00 (0.97, 1.04)	1.11 (1.02, 1.21)	
Model 3	Reference	0.99 (0.96, 1.01)	0.98 (0.95, 1.01)	1.06 (0.98, 1.16)	

Model 1: unadjusted. Model 2: adjusted for age and body mass index. Model 3: adjusted for age, body mass index, systolic blood pressure, diastolic blood pressure, and fasting plasma glucose

## Discussion

Reduced levels of serum creatinine were significantly associated with an increased risk of T2DM in men with creatinine below 1.20 mg/dl even after adjustment for age, BMI, SBP, DBP, and fasting plasma glucose. The highest category of serum creatinine levels (serum creatinine ≥1.10 mg/dl) was significantly associated with an increased risk of T2DM in women; however, this association disappeared after adjustment for age, BMI, SBP, DBP, and fasting plasma glucose.

The results for men with creatinine below 1.20 mg/dl in our study were consistent with those of previous studies in Japanese men. A study including 31,343 Japanese men without diabetes with a median observation of 7.7 years showed that low cumulative average serum creatinine levels were associated with an increased risk of diabetes after adjusting for age, smoking, BMI, hypertension, and dyslipidemia [15]. Among 8570 Japanese men aged 40–55 years at entry who had fasting plasma glucose levels < 126 mg/dl and serum creatinine levels < 2.0 mg/dl during the 4-year follow-up period, low serum creatinine was associated with an increased risk of T2DM [17]. Because we did not exclude those with comorbidities such as cardiovascular disease or cancer at baseline or those older than 65 years, other confounding factors may affect the results regarding the association between serum creatinine levels and the risk of T2DM in men with serum creatinine above 1.2 mg/dl.

Our study showed that although there was a trend that reduced levels of serum creatinine were associated with an increased risk of T2DM among women with serum creatinine below 1.1 mg/dl, the association between the level of serum creatinine and the risk of incident diabetes was not significant in women. However, there were several studies showing that the inverse association between the level of serum creatinine and the risk of incident diabetes was consistent for both sexes. In a previous study including 9667 Japanese individuals without diabetes or hypertension and with normal creatinine levels at baseline during the follow-up period (mean: 5.6 years), low serum creatinine levels independently predicted T2DM development in both men and women [13]. A Chinese cohort study including 41,439 participants (44.5% of those were women) who were ≥ 18 years (range 18–96) and did not have T2DM found that low serum creatinine levels were associated with an increased risk of T2DM after the exclusion of cardiovascular disease, cancer, and abnormally high serum creatinine levels (> 1.2 mg/dL for men and > 1.0 for women) for both men and women [14]. Because we included women with mildly elevated levels of serum creatinine (1.0–1.4 mg/dl) and did not exclude those with comorbidities such as hypertension, cardiovascular disease, or cancer, other confounding factors affecting serum creatinine may influence the results regarding the association between serum creatinine levels and the risk of incident diabetes in women.

The results regarding the association between the levels of serum creatinine and the risk of incident diabetes were different between men and women in our study. Additionally, a previous study in Korean subjects demonstrated that serum creatinine was more closely associated with the risk of T2DM in men than in women [11]. Because total muscle mass is known to be different by sex, the difference in muscle mass may affect the different results regarding the association between serum creatinine levels and the risk of T2DM between men and women. Women were reported to have lower skeletal muscle mass than men. The mean value of serum creatinine was reported to be higher in men than in women in a previous study [18].

The mechanism of the association between serum creatinine levels and the risk of incident diabetes is not clear. Several studies have demonstrated the close association between low muscle mass and dysglycemia. Among Korean subjects aged 65 or older, IR was higher in the obese group with relatively low muscle mass than in the obese group without low muscle mass [19]. Additionally, hyperinsulinemia, a compensatory response to maintain plasma glucose levels within normal ranges as an early predictor of IR, was significantly associated with loss of skeletal muscle mass in a cohort study of individuals without diabetes at the 4.6-year follow-up [20]. Increased muscle mass was associated with reduced IR and a decreased risk of diabetes [21]. The improvement of the amount of lean mass with nutritional supplements was associated with increased insulin sensitivity in elderly subjects with low muscle mass [22]. Insulin receptors in the muscle are known to play a key role in the regulation of glucose metabolism. Because skeletal muscle is the major site of insulin-mediated glucose uptake in the postprandial phase, the defect of skeletal muscle IR was suggested be the pathogenesis of the development of type 2 diabetes [23]. Increased total lean mass was associated with a decreased risk of incident diabetes for older normal-weight women [24]. Since myokines released by muscle fibers were reported to have systemic effects on the liver, adipose tissue, and pancreas function, lack of myokines such as interleukin-6 and myostatin due to reduced muscle mass may influence IR [25]. Additionally, insulin sensitizer medication use (metformin and/or thiazolidinediones) may attenuate muscle loss in men with impaired fasting glucose and diabetes [26]. Because insulin is a potent anabolic stimulus for skeletal muscle, it is possible that defects in insulin signaling can induce a reduction in muscle synthesis [27]. Additionally, glomerular hyperfiltration observed early in the natural history in patients with diabetes [28] could contribute to the association between low serum creatinine and the risk of incident diabetes mellitus. Although we did not measure the amount of ectopic adipose tissue such as visceral or epicardial adipose tissue, ectopic accumulation of adipose tissue combined with low muscle mass would affect the association between the levels of serum creatinine and the risk of incident diabetes. Further studies are needed to clarify the mechanism between the close association between serum creatinine levels and the incidence of T2DM.

Our study has several strengths. We used a larger data cohort than a previous study in the Korean population. Because the participants were recruited for health checkups at the national scale, it is reasonable to generalize the results of our study in Korea. Additionally, as women have less muscle mass than men, we analyzed subjects by sex separately.

There were some limitations. Although the measurement of serum creatinine was reliable, serum creatinine was measured in different laboratories in Korea. We did not measure the indices of IR such as homeostasis model assessment of IR due to lack of data of insulin or C-peptide. Also we did not measure sarcopenic obesity related pro-inflammatory cytokines such as interleukin 6 and tumor necrosis factor which could lead IR. Furthermore, we did not evaluate the dietary habits or physical activity that might confound the association between the levels of serum creatinine and the risk of incident diabetes. Other confounding factors, such as family history of diabetes or comorbidities that might affect the amount of muscle mass and the development of incident diabetes, were not adjusted for. Furthermore, we used serum creatinine at baseline only, and we cannot assess the relationship between the change in serum creatinine and the risk of diabetes during the follow-up period. Because our study is an observational study, it was difficult to clarify the mechanism of the relationship between serum creatinine and incident diabetes. We did not classify diabetes as type 1 or type 2 diabetes because we did not check β-cell function or islet cell autoantibodies. Asian individuals have more fat with less skeletal muscle than other ethnic groups, including European and Pacific Island adults [29, 30]. Furthermore, the risk of diabetes tended to be higher among Asian participants than among Caucasian subjects for the same categories of BMI [31]. Ethnic differences in body composition may contribute to the differences in the association between serum creatinine and the risk of incident diabetes, and it is difficult to generalize the results of our study in other ethnicities.

## Conclusions

In conclusion, lower levels of serum creatinine were significantly associated with an increased risk of T2DM in men with creatinine below 1.20 mg/dl but not in women. Therefore, measurement of serum creatinine would be useful for screening subjects with a high risk of diabetes, especially Korean men. Furthermore, these findings would be helpful to understand the heterogeneous pathophysiology of T2DM.

## Data Availability

All data generated or analyzed during this study are included in this published article.

## Abbreviations

IR: Insulin resistance
T2DM: Type 2 diabetes mellitus
BMI: Body mass index
NHIS: National Health Insurance Service
HbA1c: Glycated hemoglobin
HRs: Hazard ratios
SBP: Systolic blood pressure
DBP: Diastolic blood pressure
